# Development of a novel artificial intelligence algorithm for interpreting fetal heart rate and uterine activity data in cardiotocography

**DOI:** 10.3389/fdgth.2025.1638424

**Published:** 2025-09-16

**Authors:** Rohit Pardasani, Renee Vitullo, Sara Harris, Halit O. Yapici, John Beard

**Affiliations:** ^1^ GE HealthCare, Chicago, IL, United States; ^2^ Boston Strategic Partners, Inc., Boston, MA, United States

**Keywords:** fetal monitoring, cardiotocography, uterine activity, deep learning, computer assisted decision making

## Abstract

**Introduction:**

Cardiotocography (CTG) assesses fetal well-being through measurements of fetal heart rate (FHR) and uterine activity (UA). Manual visual assessment of fetal tracings is variable due to the subjective nature of their interpretation. Artificial intelligence (AI) using automatic signal processing may be leveraged to support consistent, comprehensive interpretations. This study demonstrated the development and training of a novel AI algorithm that analyzes and interprets certain clinical events and parameters calculated during labor to assist with clinical decisions.

**Methods:**

Fetal tracings sourced from 19 birthing centers through a US-based healthcare delivery organization were clinically interpreted, labeled, quality checked, and ratified by clinicians to be included in the study. The algorithm using deep learning and rule-based techniques was developed to identify segments of interest (accelerations, decelerations, and contractions). A three parallel one-dimensional Unet design with two inputs (FHR and UA) and one channel output each (for accelerations, decelerations, and contractions) was selected as the final architecture. Algorithm performance was evaluated through recall (sensitivity), precision, *F*1 score, and duration and numerical ratios.

**Results:**

A total of 133,696 patient files were used to create fetal tracings. After the exclusion, labeling, and ratification processes, the final datasets included 1,600 tracings for training, 421 for validation, and 591 for testing. The model provided promising performance and achieved *F*1 scores of 0.803 for accelerations, 0.520 for decelerations, and 0.868 for contractions on the final test set, with a 91.5% predicted baseline accuracy (difference of ≤5 bpm) compared to clinician interpretation.

**Conclusion:**

This study demonstrates the successful development of a novel AI algorithm utilizing FHR and UA data to analyze and interpret fetal tracing events and parameters. The algorithm may have potential to enhance patient care by supporting bedside clinician CTG interpretation.

## Introduction

Cardiotocography (CTG) is the standard practice for assessing fetal well-being (1). Fetal distress, which may require medical intervention, is diagnosed using baseline fetal heart rate (FHR), changes from the baseline, and its reaction to uterine contractions as labor progresses (2). Manual visual assessment of fetal tracings requires expertise, and interpretation may vary based on clinical experience leading to inter- and intra-observer disagreement (1). The National Institute of Child Health and Human Development (NICHD) guidelines (3) were developed to provide standardized terminology and classification systems to reduce subjectivity and variability in CTG interpretation. While the guidelines improve consistency in clinical decision-making and communication, subjectivity is not eliminated, particularly in interpreting ambiguous tracings where clinical judgment varies widely [e.g., indeterminate (Category II) FHR patterns]. There is a need for an objective and consistent system for interpreting CTG data to optimize maternal and neonatal outcomes.

Computer-based systems for interpreting CTG offer potential solutions, and rule-based/programmatic algorithms have been developed to calculate FHR baseline and detect accelerations/decelerations. However, a study (4) comparing 11 existing algorithms concluded that none achieved clinical expert level of assessment. To address the existing performance gap, artificial intelligence (AI) deep-learning methods have been proposed to improve automated CTG interpretation through advanced waveform analysis and pattern recognition. Deep learning AI applied to CTG interpretation has primarily focused on convolutional neural networks (CNNs), recurrent neural networks, and hybrid architectures, aiming to reduce high inter- and intra-observer variability and improve diagnostic accuracy for fetal compromise (5–12). Some studies have implemented multi-branch networks to process heterogeneous data types (e.g., images and clinical parameters), while others have applied domain adaptation techniques to improve generalizability across different clinical sites (7, 11). One-dimensional (1D) CNNs have been used to capture temporal patterns in FHR and uterine activity (UA) signals, and attention mechanisms have been introduced to enhance feature extraction (12). Despite these advances, previously developed deep-learning algorithms have generally focused solely on FHR without integrating UA signals, addressed classification tasks rather than event segmentation, or have not explored the use of parallel U-Net branches for CTG interpretation (1, 9, 13–17). Excluding the ongoing impact of UA on FHR during labor removes an essential clinical element used to assess fetal well-being and formulate the delivery plan. Leveraging AI that processes both FHR and UA data can provide a more comprehensive and insightful representation of maternal and fetal well-being and may enhance CTG interpretation (15). An algorithm examining event segmentation enables a more detailed and clinically informative analysis of maternal and fetal status, allowing for the identification of specific physiological events (e.g., accelerations and decelerations) in addition to overall FHR patterns. While U-Net architectures are widely used for segmentation tasks in biomedical imaging, their adaptation to 1D time series, combined with the use of multiple parallel branches, offers the ability to simultaneously extract distinct temporal features from different signal modalities or segments. This design enables multi-label event segmentation, allowing for the detection of overlapping physiological events (e.g., decelerations occurring during contractions) and represents a methodological advance over existing single-branch or hybrid models (7, 9–12).

To our knowledge, there is no previously-disclosed AI algorithm utilizing both deep learning and rule-based that performs these functions through a parallel 1D U-Net design with FHR and UA data to describe events, parameters, and information about fetal tracings. This study aimed to demonstrate the deep learning and rule-based development (18) of a novel AI algorithm (19), which analyzes and interprets FHR and UA clinical data to support clinician decision making during the progression of labor.

## Methods

### Data sourcing and pre-processing

**Figure 1:**
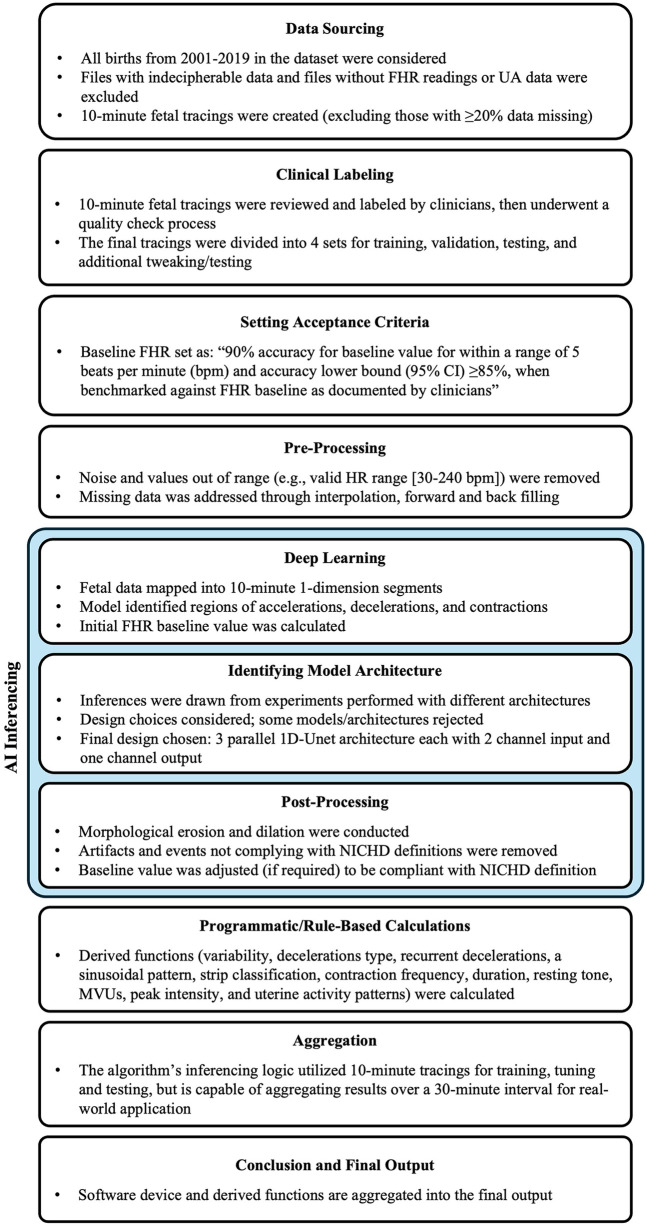
Study design. FHR, fetal heart rate; MVU, Montevideo units; NICHD, National Institute of Child Health and Human Development; UA, uterine activity. Study design for algorithm development.

Fetal tracings were sourced from a US-based healthcare delivery organization that included 19 separate birthing centers. The de-identified dataset included delivery data in patient records from 2001 to 2019 (Figure 1). In total, 222,169 patient files were identified, with each file corresponding to a unique patient/maternity case. All available raw FHR and UA waveform data were received and processed by a US Food and Drug Administration (FDA)-cleared and Conformité Européene (CE)-marked electronic maternal-fetal monitor (e.g., Corometrics 116, 120, 170, or 250 series, GE HealthCare, Chicago, IL, USA; HP135x, Hewlett Packard, Palo Alto, CA, USA). FHR samples were received at a frequency of 4 Hz and recorded as beats per minute (bpm). Uterine contraction samples, including an estimation of uterine pressure from 0 to 127 mmHg, were received at the same frequency (4 Hz) from either an internal uterine pressure (IUP) catheter or an external pressure-sensitive contraction transducer (tocodynamometer).

The raw data underwent pre-processing to prepare for modeling, removing noise and values out of range [e.g., valid heart rate range (30–240 bpm)], and addressing minor gaps and missing values through interpolation, forward and backward filling. One 10-minute fetal tracing from each patient file with FHR and UA data was clipped for inclusion in the study based on the quality of the recording. The tracing exclusion criteria were: (1) multiple gestation pregnancies; (2) files that could not be read or did not have both FHR readings and UA data available; and (3) fetal tracings with ≥20% missing data.

### Data preparation

The fetal tracings were processed to create final datasets for training, tuning (validating), and testing the algorithm. Qualified clinicians, defined as a registered nurse (RN) in obstetrics and gynecology who had ≥5 years of experience as a practicing RN in a labor and delivery or obstetrics and gynecology department and was trained on the NICHD guidelines (3), reviewed and labeled the tracings. The quantity of tracings included was determined based on the availability of clinical experts, the given timeframe of the study, and the frequency of events as described by NICHD guidelines (3). Labeling included identification of the baseline value, and regions of accelerations, decelerations, contractions, and variability. Tracings that included artifacts that precluded the ability to interpret a continuous 10-minute tracing were excluded from clinician labeling but utilized to train the algorithm's “noise filter”.

Four sets of tracings were created from the labeled database: the training, tuning (validation), gold standard test, and additional test sets. All tracings underwent a ratification process requiring three clinical expert opinions (either accepting or rejecting the interpretation of the labels) for all included labeled tracings: one from the clinician who initially interpreted/labeled the tracing, and two additional, independent opinions from the clinicians who ratified the interpretations. The training, validation, and additional test sets all had at least one independent reviewer acceptance of the initial labeled interpretation during ratification. The training set was used to train the deep learning model. The validation set was used to fine-tune the model's hyperparameters (e.g., loss function, batch size, epochs, optimizer, learning rate, and momentum) and assess its performance during the development and training process. The gold standard test set, included tracings which both independent reviewers accepted the initial interpreted labels, was not used during the model development and tuning process but provided an unbiased evaluation of the trained model's performance. The rejected labels, in which both clinicians rejected the labeled interpretations during ratification, were not used to train, tune, or test the algorithm. A group of tracings (the additional set) was set aside in case additional performance testing/tweaking is needed.

Data quality checks were applied to the tracings during labeling and ratification processes. Post-processing ensured there was only one discrete baseline value, and utilized morphological erosion and dilation to join segments that were extremely close to each other as well as removed segments too small to qualify as accelerations/decelerations/contractions as per the NICHD definitions (3).

### Algorithm development

The algorithm was trained, validated, and tested using deep learning and rule-based techniques. A final model architecture was selected based on a thorough evaluation of multiple approaches. To generate outputs, the algorithm analyzed and interpreted events, parameters, and values typically calculated by clinicians during the labor and delivery clinical workflow. Algorithm performance was evaluated through metric outputs, which served as a comparison to those from clinical experts.

#### Algorithm targets

FHR baseline was defined based on the NICHD guidelines as the mean FHR during a 10-minute segment, rounded to increments of 5 bpm (3). FHR data was mapped into 10-minute one-dimensional (1D) waveform segments. Segments of interest in FHR tracings, including accelerations, decelerations, and contractions, were identified using deep learning before calculating a baseline. Accelerations and decelerations, periods of marked variability, or baseline segments that differed by >25 bpm were excluded from the FHR baseline determination. Based on a reference study (4) and multiple iterations of the model-building algorithm, the preliminary internal target for the FHR baseline calculation was determined to be 90% accuracy of the baseline value within a range of 5 bpm and accuracy lower bound (95% CI) ≥85%, when benchmarked against FHR baseline as documented by qualified clinicians.

#### Model architecture

The deep learning model was trained using a weighted Dice loss function, balancing acceleration, deceleration, and contraction. Training was conducted for 2,000 epochs with a batch size of 32. Stochastic Gradient Descent was used as the optimizer, with a learning rate of 0.5 and no momentum. The model had 56,667 total parameters, with most (56,115) being trainable.

**Figure 2:**
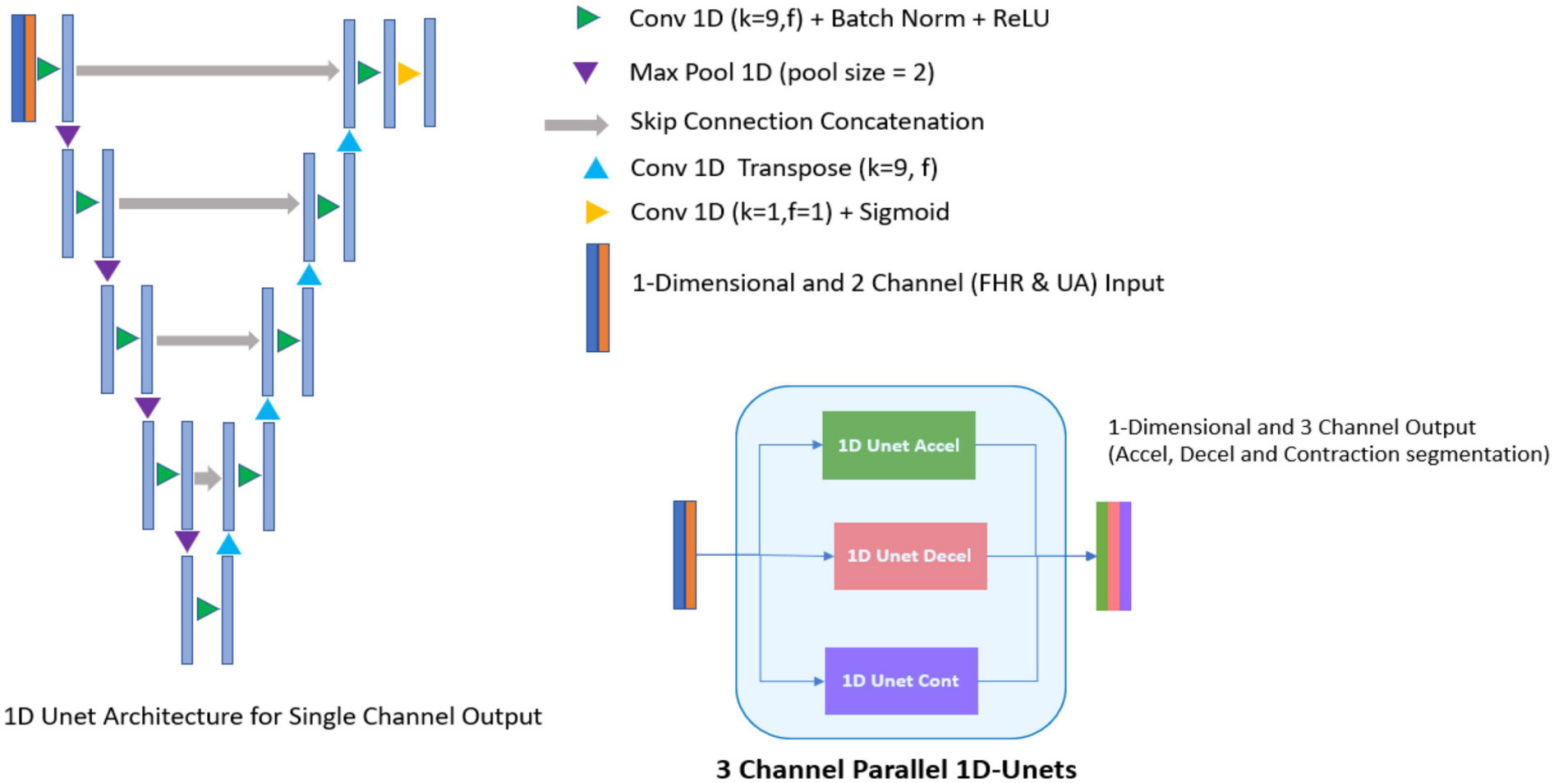
Deep learning model architecture (3 channel parallel 1D-unets). Experiments showed that three parallel 1D-Unet model architecture with two channel inputs (fetal heart rate and uterine activity) and one channel output each (for accelerations, decelerations, and contractions) was the best design choice for the algorithm.

**Figure 3:**
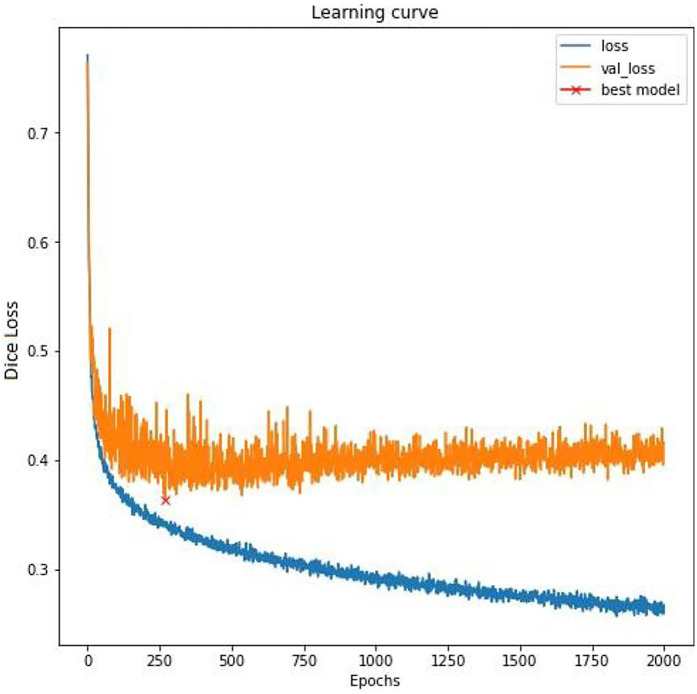
Training curve. A fluctuating training loss and highly fluctuating validation loss is indicative of subjectivity in labeling, which is typical in the assessment of fetal tracing. To mitigate the overfitting to the noisy training labels and to ensure better generalization to unseen data, the model with the best validation loss was saved (a strategy known as early stopping).

Experiments were performed using different architectures and configurations to identify the best-fitting model that is able to address the unique clinical challenges targeted by the algorithm. Specifically, the potential models needed to look at both the left and right of the waveform while segmenting, rather than just one direction, to be able to accurately identify an FHR baseline. In addition, the analytics required the segmentation of three events (accelerations, decelerations, and contractions) with two input channels (FHR and UA). Large models, sequence to sequence bi-directional long short-term memory (LSTM) network which processes the waveform as a sequence over time and 1D-Unet which detects patterns and structures in waveform data, were approaches that fit the requirement. The following inferences were drawn during architecture assessments: (1) large models tended to overfit (i.e., learn too much from the training data without generalizing well) due to an inherent lack of agreement among clinical experts about the definition of events; and (2) experiments showed that 1D-Unet architecture performed better than bi-directional LSTM. Ultimately, a three parallel 1D-Unet with two channel inputs (FHR and UA) and one channel output each for accelerations, decelerations, and contractions was chosen as the most appropriate design (Figure 2). The model with the best validation loss was saved and used for inference on the test set (Figure 3).

#### Algorithm output

**Figure 4:**
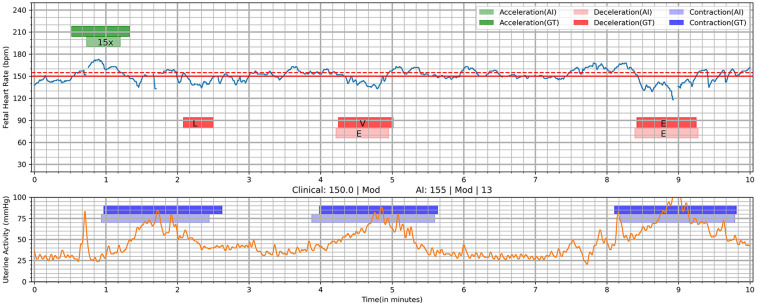
Visualization of deep learning Algorithm's output on a 10-min tracing. AI, AI algorithm; E, early; GT, ground truth (clinician interpretation); L, late; V, variable; 15×, 15 × 15 acceleration. Deep learning was utilized to identify segments of interest including acceleration, deceleration, and contractions. This shows the interpretations of the AI algorithm compared to clinician interpretation (ground truth). Clinical: 150.0 | Mod, <FHR baseline as marked by clinicians (ground truth)> | <Variability by clinicians>; AI: 155 | Mod | 13, <FHR baseline by AI > | <Variability by AI > |.

The algorithm used deep learning techniques for software device functions and rule-based algorithms for derived functions to generate outputs (Table 1). Software device functions were directly dependent on (calculated using) the deep learning algorithm and were clinically fundamental to the assessment of fetal tracings (Supplementary Table S1). Derived functions were mathematical calculations on the values obtained from software device functions (3, 20). The output values, parameters, and events were calculated by the algorithm once per minute based on the last 10 min of FHR and UA data. A minimum sample size of 10 min was required for the algorithm assessment and output (Figure 4). While the algorithm's inferencing logic utilized 10-minute tracings for training, validating, and testing, the algorithm is capable of aggregating results over a 30-minute interval to enable seamless and continuous inference over extended durations for real-world application since low risk patients generally require 30-minute assessments (21, 22). Notably, the development also supports future adaptation for 15-minute inferences to address the assessment of high-risk patients (21, 22).

**Table 1:** Algorithm outputs.

Functions	Output values
Software device	• FHR baseline • Accelerations • Decelerations • Contraction region identification
Derived	• Variability of FHR baseline • Deceleration type(s): early, late, prolonged, variable, or undefined • Contraction frequency • Contraction duration • Sinusoidal pattern • NICHD tracing or strip classification [Category I (normal), II (indeterminate), or III (abnormal)] (Supplementary Table S2) • UA pattern: tachysystole • FHR patterns: tachycardia, bradycardia • Recurrent decelerations • Resting tone (IUP only) • MVUs (IUP only) • Peak intensity (IUP only)

FHR, fetal heart rate; IUP, internal uterine pressure; MVU, Montevideo units; NICHD, National Institute of Child Health and Human Development.

#### Evaluation metrics

The primary performance evaluation metrics were recall (sensitivity), precision, and *F*1 score. Formulas for calculating evaluation metrics can be found in Supplementary Table S4. Due to a segment-wise approach, an instance of a predicted event that overlapped with the same event in ground truth (as labeled by clinicians) was taken as a true positive. *F*1 score provided a balanced measure of a model's performance by considering both the ability to identify relevant results (recall) and the accuracy of those identified results (precision). Secondary performance metrics included duration ratios and numerical ratios that were analyzed to ensure that events were not over- or underestimated. Duration ratio was calculated to showcase the algorithm's accuracy in detecting the correct length of segments of interest, while numerical ratio was calculated to ensure algorithm's prediction accuracy of the number of segments of interest.

## Results

**Figure 5:**
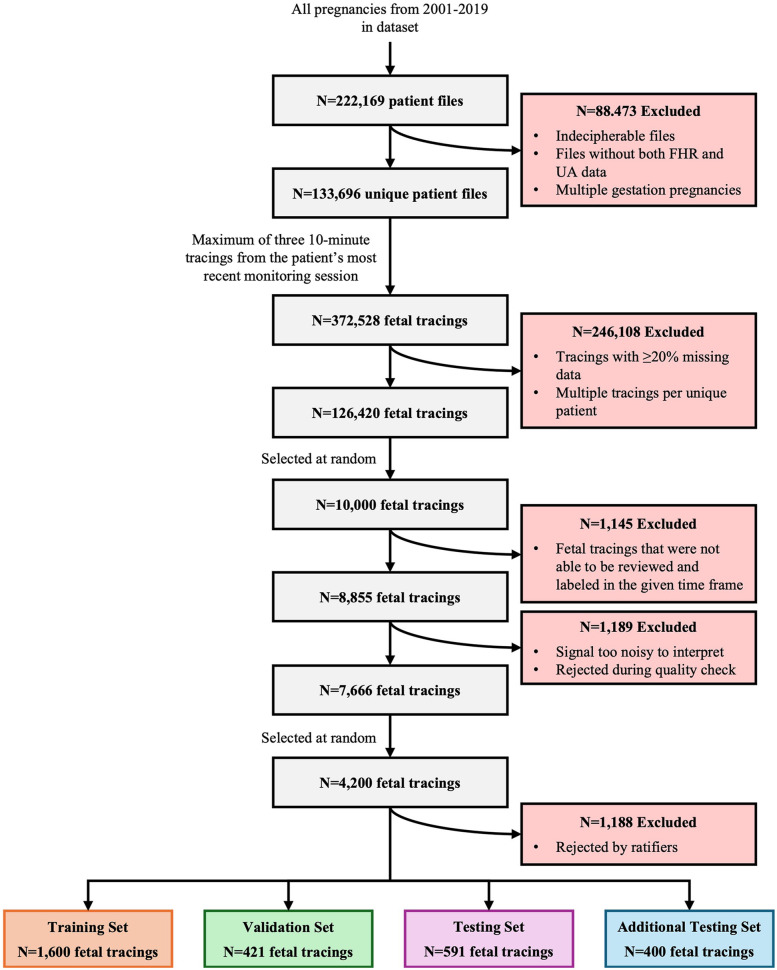
Study attrition. This figure illustrates the systematic attrition of FHR and UA values extracted from de-identified patient files from 2001 to 2019, detailing the number of exclusions at each stage of data processing, quality control, and review. It began with 222,169 unique patient files, progressively narrowing down the dataset based on various exclusion criteria (e.g., incomplete data, poor signal quality, expert rejection) to arrive at a final set of 4,200 high-quality fetal tracings. These tracings are then allocated into distinct training, validation, testing, and additional testing sets for analysis.

A total of 222,169 files were reviewed for this program, resulting in the inclusion of 133,696 patient records with 372,528 tracings (Figure 5). After excluding tracings with considerable data missing and multiple tracings per unique patient, 126,420 fetal tracings were included in the study. Of these, 10,000 10-minute tracings were randomly extracted for labeling. Within the timeframe allotted for the task, clinicians completed the review and labeling of 8,855 fetal tracings, 1,189 of which were rejected due to poor quality and noisiness, resulting in 7,666 tracings that were accepted. Ratification of clinical labeling occurred in a randomly selected 4,200 fetal tracings. Of these, 1,188 tracings were rejected by the ratifying clinicians, 2,421 were accepted by at least one of the two independent reviewers, and 591 were accepted by both independent reviewers. Thus, the final datasets included 1,600 tracings for the training set, 421 for the validation set, 591 for the gold standard test set, and 400 for the additional testing set.

The model achieved *F*1 scores of 0.803 for accelerations, 0.520 for decelerations, and 0.868 for contractions on the test set (Table 2). In the test set, 91.5% of predictions had a difference of ≤5 bpm compared to the ground truth (Table 3). Predictions of a baseline value (when ground truth was no baseline) were seen in 1.7% (*n* = 10) of test set tracings, and predictions of no baseline value (when ground truth was a value) were seen in 0.3% (*n* = 2) of test set tracings. Two tracings (*n* = 2; 0.3%) demonstrated agreement between ground truth and the model when no true baseline was predicted.

**Table 2:** Model performance.

Model outputs	Training set	Validation set	Test set
Accelerations			
Recall	0.688	0.665	0.736
Precision	0.824	0.813	0.883
Dur Ratio (Pred/True)	0.936	0.861	0.852
Num Ratio (Pred/True)	0.838	0.814	0.828
*F*1 Score	0.750	0.732	0.803
Decelerations			
Recall	0.546	0.549	0.460
Precision	0.708	0.641	0.598
Dur Ratio (Pred/True)	0.923	1.008	0.811
Num Ratio (Pred/True)	0.777	0.884	0.750
*F*1 Score	0.617	0.591	0.520
Contractions			
Recall	0.893	0.866	0.935
Precision	0.828	0.827	0.810
Dur Ratio (Pred/True)	1.037	0.984	1.072
Num Ratio (Pred/True)	1.087	1.082	1.166
*F*1 Score	0.859	0.846	0.868

Dur Ratio, duration ratio; Num Ratio, numerical ratio; Pred, predicted.

**Table 3:** Ground truth vs. Algorithm baseline.

Interpretation comparison	Training set		Validation set		Test set	
	Count	Fraction	Count	Fraction	Count	Fraction
Difference						
0 bpm	725	0.453	177	0.42	306	0.518
5 bpm	717	0.448	202	0.48	235	0.398
≤5 bpm	1,442	0.901	379	0.9	541	0.915
10 bpm	89	0.056	16	0.038	23	0.039
15 bpm	18	0.011	12	0.029	3	0.005
20 bpm	6	0.004	0	0.0	6	0.01
Agreement and disagreement						
g:NB, p:Val^a^	25	0.016	5	0.012	10	0.017
g:Val, p:NB^a^	6	0.004	3	0.007	2	0.003
g:NB, p:NB^a^	0	0.0	0	0.0	2	0.003
Other	14	0.009	6	0.014	4	0.007
Total	1,600	1.0	421	1.0	591	1.0

^a^ g:NB, ground truth (clinician interpretation) is no-baseline; p:Val, AI prediction is a value; g:Val, ground truth is a value; p:NB, AI prediction is no-baseline.

The development process included specific performance benchmarks to meet real-time monitoring requirements in clinical environments, and as such, to operate on standard healthcare information technology (IT) infrastructure. The algorithm achieved end-to-end interference for a single 10-minute CTG tracing in <500 ms, meeting the real-time processing requirements for bedside monitoring. Additional details on the performance characteristics can be found in Supplementary Table S4.

## Discussion

This novel AI algorithm was designed to analyze and interpret specific events, parameters, and values that clinicians assess during the labor and delivery process. To our knowledge, there is no AI algorithm that performs these same functions on FHR and UA data. This AI algorithm was trained using fetal tracings that were clinically labeled by qualified clinicians. Through this training and after evaluating various algorithms’ performance, the most appropriate model architecture was identified as a three parallel 1D-Unet design, with two channel inputs (FHR and UA) and one channel output each for accelerations, decelerations, and contractions. The model demonstrated encouraging performance in the assessment of FHR and UA when compared to expert clinical reviewers.

The model provided promising results measured as recall, precision, and *F*1 scores for accelerations, decelerations, and contractions within the test set, and showed a 91.5% predicted baseline accuracy (difference of ≤5 bpm) compared to clinician interpretation. A difference of 5 bpm is consistent with clinical practice to round measurements to the nearest 5 bpm. Clinician and algorithm interpretation differed slightly when debating cases of no baseline value. Importantly, the algorithm was engineered to determine the most plausible baseline within its predefined rules based on any amount of data it received, minimizing noise through filtering techniques. This approach may be preferable, especially in cases of ambiguity, where clinicians may adopt a cautious or conservative approach when visually assessing a baseline and refrain from assigning a baseline. The differences in these approaches may help to explain the instances where the algorithm identifies a baseline, while clinicians do not. While the algorithm is not designed to replace clinical interpretation, it may serve as a supportive reference and resource when traditional methods of calculating baseline values are not fully conclusive.

Meticulous adherence to the foundational principles of responsible AI (validity, reliability, transparency, accountability, privacy, safety, robustness, interpretability, and fairness) was followed during the development and training of this algorithm to ensure ethical development and deployment (23). The model was configured to systematically document tracing events every minute, enabling users to trace individual outputs to specific points in time, while also avoiding making inferences from tracing data with significant gaps. A “human-in-the-loop” approach was taken to allow clinicians to override the AI's interpretation when necessary. The algorithm's development involved continuous clinician feedback to maintain consistency with clinical needs and adherence to ethical standards. Every output was made traceable to the log and supported by clinical rationale, reinforcing accountability. The outputs were also designed to be reliable and accurate, as well as interpretable by clinicians with varying levels of expertise, through its handling of missing data and assured alignment with NICHD standards (3). Patient privacy was prioritized, personally identifiable information was protected, and all development and testing were conducted using anonymized data to ensure confidentiality.

This algorithm aimed to facilitate unbiased care, equitable access to high-quality fetal monitoring, and address disparities in maternal and neonatal care. Disparities in healthcare may exist among the significant differences in access to quality medical care experienced by individuals depending on where they reside, their income level, and their racial or ethnic background (24). Further, these disparities often disfavor marginalized populations, resulting in poorer health outcomes due to factors such as limited resources and access to care, inability to afford proper care, and potential implicit bias within the healthcare system (25, 26). The algorithm may reduce disparities by equipping clinicians with precise, objective, and real-time decision-support tools, supplementing existing technologies, to reduce care variability and enable timely, informed interventions for improved maternal and fetal outcomes. AI-driven, real-time clinical decision support systems have shown potential for improving care quality by standardizing recommendations, providing actionable insights, and reducing decision-making burdens (27–29). This tool addresses a pressing clinical need stemming from the variability of clinical judgment and care. The AI algorithm's output is designed to be interpretable by clinicians of varying expertise, offering consistent, real-time objective assessments that may reduce the variability of inter- and intra-observer interpretation. While not replacing the bedside care, the FHR AI algorithm may be a valuable clinical decision support tool that can simplify clinician workload, reduce repetitive manual tasks, and enhance efficiency; all of which may address clinical burnout.

The algorithm demonstrated the integration of cutting-edge machine learning with clinical applications, offering a development framework for future innovations and showcasing the potential of AI in obstetrics. Although the underlying algorithm architecture was based on U-Net, the novelty of our approach lies in its adaptation, application, and architectural enhancements for CTG waveform segmentation, being a domain in which such methods have not previously been demonstrated. Specifically, a parallel 1D U-Net design enabling simultaneous (multi-label) segmentation of multiple event types directly from raw FHR and UA signals. This multi-label capability, the ability to assign more than one event label to a given time point and capture overlapping physiological events, combined with domain-specific post-processing and event refinement, differentiates this method from prior applications of U-Net in the waveform domain. To our knowledge, no previous studies have employed 1D U-Net for multi-label segmentation of CTG waveforms at this level of granularity and clinical relevance. This development technique may inform future studies to address unmet needs within patient care. Model drift and degradation were heavily considered during the development process and remain unlikely events for a few reasons. Following the standards and guidelines established by NICHD (3) and the Association of Women's Health, Obstetric, and Neonatal Nurses (AWHONN) (20), this algorithm was created to be a fixed model that does not dynamically learn in the field, and therefore, model drift is unlikely. Despite being non-retrainable in nature, the algorithm does not rely on device-specific metadata and data types/formats remain consistent across technologies, thus the algorithm's ability to interpret fetal waveforms would not be affected by the introduction of new sensor technologies. Because of this and since the model was selected based on its performance to mitigate overfitting risk, model degradation is not expected.

Technological advances and clinician support through this algorithm may also serve to improve the professional experience of bedside clinicians. The increasing care burden, documentation requirements, and decision fatigue placed on clinicians contribute significantly to burnout (30, 31). Burnout is a serious occupational hazard among clinicians, which not only impacts clinician well-being but also raises the risk of errors and missed patient care processes (30–34). The workflow support of FHR parameter measurement and pattern recognition may serve to reduce time consuming repetitive manual tasks and increase clinician availability for more rewarding elements of patient care. Ideally, technology solutions such as this algorithm may shift an increased portion of clinician activities to focus on the patient and family members and increase professional satisfaction.

The future integration of this AI algorithm into clinical workflows holds promise for improving maternal and neonatal outcomes; however, continued evaluation of its safety, fairness, and inclusiveness, as well as ongoing collaboration between clinicians and AI developers, will be essential to ensure ethical implementation and widespread adoption. By addressing key challenges such as inter-observer variability and the subjectivity inherent in traditional FHR assessment, the algorithm offers a reliable tool for identifying fetal distress and supporting timely clinical decision-making. Furthermore, the model's robust performance across diverse datasets underscores its applicability in real-world clinical settings and its ability to promote equitable care.

There are inherent limitations to fetal monitoring algorithm development and clinical adoption. First, fetal tracing analysis involves a high degree of subjectivity. Among the parameters evaluated—baseline, accelerations, decelerations, and contractions—decelerations are particularly subjective, often leading to significant disagreement among clinicians (35, 36). This variability poses a challenge for learning consistent patterns, especially when the training data is labeled by different clinicians with varying interpretations. Consequently, this subjectivity represents an inherent limitation in computational models for fetal analytics, reflecting the broader challenges within the domain. Second, the successful adoption of AI depends on factors like explainability, usability, and seamless integration into existing healthcare processes, as well as addressing barriers like clinician skepticism and workflow interruptions. Further refinement of AI systems is necessary to address these concerns. Despite achieving strong performance metrics (e.g., high sensitivity and specificity, real-time capability), this approach, similar to other AI-based CTG analysis systems, has not yet been validated in large-scale, prospective, multicenter clinical studies. The absence of such validation limits the immediate generalizability of these findings and currently precludes widespread clinical adoption or guideline endorsement. Future work should focus on (1) evaluating algorithm performance on larger, more diverse, and prospectively collected datasets to ensure data quality and reduce selection bias; (2) conducting multicenter trials to assess generalizability across different clinical settings, populations, and acquisition systems; and (3) investigating pathways for integration into routine practice, including considerations of workflow compatibility, clinician trust, and regulatory approval processes.

## Conclusion

This study presents the successful development of a novel AI algorithm utilizing FHR and UA data to analyze and interpret fetal tracing events and parameters. The algorithm has the potential to enhance the accuracy and consistency of CTG interpretation, reduce disparate healthcare outcomes, and support bedside clinician workflows. This effort represents a significant step toward leveraging AI to enhance clinical decision-making and address longstanding challenges in obstetric care, advancing the quality of perinatal medicine.

## Data Availability

The data analyzed in this study is subject to the following licenses/restrictions: Due to the legal/commercial nature of the research, supporting data is not available. All necessary data has been disclosed. Requests to access these datasets should be directed to John Beard, john.beard@gehealthcare.com.
