# Supplementary material for: Development of a novel artificial intelligence algorithm for interpreting fetal heart rate and uterine activity data in cardiotocography
Source: Front Digit Health. 2025 Sep 16;7:1638424. doi: 10.3389/fdgth.2025.1638424 (PMC12479546; doi:10.3389/fdgth.2025.1638424)
Supplement: Supplementary file 1 [file Datasheet1.docx]

# **Supplement**

### **Supplemental Table 1.** Algorithm Outputs Calculation Methodology

| **Item** | **Calculation methodology** |
| --- | --- |
| **Software Device Functions** | |
| Fetal Heart Rate Baseline | Identified regions of Accelerations, Decelerations, and Marked Variability using deep learning derived algorithm and then calculating the mean of values in the remaining regions. At least 2 minutes of data must be available. Calculations adhere to NICHD definition (1) |
| Accelerations | Acceleration region identification: Deep Learning algorithm to detect a visually apparent abrupt increase in FHR with programmatic post-processing to ensure adherence to NICHD definition (1)  Output labeling for 10x10, or 15x15 as per NICHD definitions (1) |
| Decelerations: Detecting regions of Decelerations | Deceleration region identification: Deep Learning algorithm to detect a visually apparent, usually symmetrical, gradual decrease in FHR and return to baseline with programmatic post-processing to ensure adherence to NICHD definition (1) |
| Contraction region identification | Identify regions of contractions using Deep Learning. A programmatic post-processing is performed to ensure that identified regions are compliant to NICHD guidelines (1).  The clinically relevant and derived parameters from contraction identification are frequency and duration below. |
| **Derived Functions** | |
| Variability of FHR baseline | Programmatically calculated with a rule-based algorithm matching definitions of Absent, Minimal, Marked, or Moderate stated in the NICHD definitions(1)  NICHD Definition for Variability: “Determined in a 10-minute window, excluding accelerations and decelerations. Fluctuations in the baseline FHR that are irregular in amplitude and frequency and are visually quantified as the amplitude of the peak-to-trough in bpm.”  Mathematical/Programmatic calculation logic:   - Exclude regions of accelerations & decelerations - Divide the remaining region in smaller segments - Calculate the difference in maxima and minima for each segment (segment variability) - Take the median value of the range of segment variability values obtained |
| Deceleration Type (s): Early, Late, Prolonged, Variable, or Undefined | Deceleration type, derived from Decelerations: Programmatically derived value based on the NICHD “Characteristics of Decelerations” table (1). Output values are Early, Late, Prolonged, Variable, or Undefined |
| Contraction Frequency | Contraction region identification (software device function) is followed by programmatic calculation to determine time interval between start of every two consecutive contractions. The shortest and longest values among these intervals are reported (e.g. 3 – 5 minutes) for the assessed time-period. |
| Sinusoidal FHR pattern | Programmatically calculated over a period of 20 minutes with a rule-based pattern detection algorithm to identify a sine wave–like undulating pattern as explained in the NICHD guidelines (1).  NICHD Definition: “Visually apparent, smooth, sine wave-like undulating pattern in FHR baseline with cycle frequency of 3-5/minute that persists for ≥20 minutes.”  Mathematical or programmatic method used: A rules-based algorithm was developed based on existing sinusoidal patterns provided by NICHD, and the output of the algorithm was validated by clinicians.  Sinusoidal patterns are typically found in 0.004% of the fetal tracings or strips (1, 2) |
| NICHD Tracing or strip classification: Category I, II, III | Programmatic calculation using parameter inputs as defined in “Three-Tier Fetal Heart Rate Interpretation System” from the NICHD guidelines (1) (**Supplemental Table 2**) |
| UA Pattern: Tachysystole | Programmatic calculation for Tachysystole matching definition provided in the NICHD guidelines (1) indicating more than 5 contractions in 10 minutes, averaged over a 30-minute time interval |
| FHR Patterns: Tachycardia, Bradycardia | Programmatic calculation matching definition provided in the NICHD guidelines (1) for Tachycardia or Bradycardia of FHR baseline.  Tachycardia: Baseline Rate >160 bpm  Bradycardia: Baseline Rate <110 bpm |
| Recurrent Decelerations | Programmatic calculation matching definition provided in the NICHD guidelines (1), with late or variable decelerations occurring ≥50% of contractions in any 20-minute window. |
| Resting Tone (IUP only): | Derived programmatic calculation, indicating the pressure value of the uterus when not contracting. This is derived as a mean value of uterine pressure values excluding the contraction regions. This calculation matches the definition described in AWHONN standards. The value is only displayed for IUP indicated data. |
| MVUs (IUP only): | Montevideo Unit calculation calculated by taking the peak intensity or amplitude (in mmHg) for each contraction occurring in a ten-minute window of time and adding the numbers together. This calculation matches the definition described in AWHONN standards. This value is only reported for IUP indicated data. |
| Peak Intensity (IUP only): | Minimum and Maximum value in mmHg received for identified contractions. This calculation matches the definition described in AWHONN standards (3). The value is only reported for IUP indicated data. |

Abbreviations: AWHONN, Association of Women’s Health, Obstetric, and Neonatal Nurses; FHR, fetal heart rate; IUP, intrauterine pressure; NICHD, National Institute of Child Health and Human Development; MVU, Montevideo unit

**References:**

1. Simpson KR. NICHD Definitions and Classifications: Application to Electronic Fetal Monitoring Interpretation. 2010.

2. Jackson M, Holmgren CM, Esplin MS, Henry E, Varner MW. Frequency of fetal heart rate categories and short-term neonatal outcome. Obstet Gynecol. 2011;118(4):803-8.

3. Lyndon A, Wisner K. AWHONN Fetal Heart Monitoring: Principles and Practices: Kendall Hunt Publishing Company; 2021.

**Supplemental Table 2.** Fetal Heart Rate Pattern Classification and Interpretation

| **Category** | **Interpretation** | **Features** |
| --- | --- | --- |
| **I**  **Normal** | Tracings in this category are strongly predictive of normal acid-base status at the time of observation. | - Baseline rate 110 to 160 beats per minute - Baseline variability moderate - No late or variable decelerations - Early decelerations present or absent - Accelerations: present or absent |
| **II**  **Intermediate**  All tracings not categorized as Category I or III. May represent many tracings that are encountered in everyday clinical practice. | Tracings in this category are not predictive of abnormal acid-base status, however there are insufficient data to classify them as either Category I or Category III. | - Baseline rate: Bradycardia not accompanied by absent baseline variability - Baseline rate: Tachycardia - Minimal variability - Absent variability without recurrent decelerations - Marked variability - Absence of induced accelerations after fetal stimulation - Recurrent variable decelerations with minimal or moderate variability - Prolonged deceleration - Recurrent late decelerations with moderate variability - Variable decelerations with “slow return to baseline,” “overshoots” or “shoulders” |
| **III**  **Abnormal** | Tracings in this category are predictive of abnormal acid-base status at the time of observation. | - Absent variability and any of the following: - Recurrent late decelerations - Recurrent variable decelerations - Bradycardia - Sinusoidal pattern |

**Supplemental Table 3.** Dataset Distribution and Event Frequency

| **Set** | **Tracings** | **Accelerations** | **Decelerations** | **Contractions** |
| --- | --- | --- | --- | --- |
| **Acceleration, Deceleration and Contraction Events in Each Set** | | | | |
| Training | 1600 | 2567 | 700 | 3409 |
| Validation | 421 | 663 | 173 | 920 |
| Test | 591 | 992 | 176 | 1063 |
| **Derived Functions** | | | | |
| Training | 1600 | 1.60 | 0.44 | 2.13 |
| Validation | 421 | 1.57 | 0.41 | 2.18 |
| Test | 591 | 1.68 | 0.30 | 1.80 |

**Supplemental Table 4.** Evaluation Metric Formulas

| **Formula Type** | **Formula** |
| --- | --- |
| Precision^1^ | TP / (TP + FP) |
| Recall^1^ | TP / (TP + FN) |
| F1 Score | (2*Precision*Recall) / (Precision + Recall) |
| Duration Ratio | $\frac{Sum of durations of all events of particular type in AI Output}{Sum of durations of all events of that type in Ground Truth}$ |
| Numerical Ratio (or Event Count Ratio) | $\frac{Number of all events of particular type in AI Output}{Number of all events of that type in Ground Truth}$ |

**Abbreviations:** AI, artificial intelligence; TN, true negative; TP, true positive; FN, false negative; FP, false positive

^1^ While iterating over all AI-detected events, an AI event sample was considered true positive (TP) if it had 5 seconds or more overlap with ground truth (GT) event, else that AI event sample was considered false positive (FP). We then iterated over all GT events. A GT event sample was considered TP if it had 5 seconds or more overlaps with AI event, otherwise that GT event sample was considered false negative (FN).

**Supplemental Table 5.** Computation time and Performance Characteristics

| **Metric** | **Specification** | **Achieved Performance** |
| --- | --- | --- |
| Processing Capacity | 100 beds per minute | 100 beds in ~7.6 seconds |
| CPU Utilization | - | ~16% |
| Memory Utilization | - | ~81% |

Abbreviations: CPU, central processing unit
